# National Liberal Women’s Suffrage Association

**Published:** 1890-02

**Authors:** Matilda Joslyn Gage

**Affiliations:** Fayetteville, N. Y.


For HALL'S JOURNAL OF HEALTH.

### NATIONAL LIBERAL WOMEN'S SUFFRAGE ASSOCIATION.

### TO LIBERAL WOMEN.

The time has arrived for a national organization of liberal-thought women who can work unitedly against the forces now opposing freedom. Women's enfranchisement progresses but slowly from various causes—one, the growing conservative tendency of existing societies. An association of brave, far-seeing women is needed who will dare point out the tendency of the teachings of the church to hold women both in political and religious subjection. Again, the church party in politics, composed of both Catholics and Protestants, was never as defiant, never as sure of success as at present, its aim being union of church and State.

A convention for the purpose of canvassing these questions will be held in Washington, February 24th and 25th, 1890. Women interested are invited to correspond with

MATILDA JOSLYN GAGE,
Fayetteville, N. Y.

To whom all contributions in and for this purpose may be sent.

